# The roles of glucagon-like peptide-2 and the intestinal epithelial insulin-like growth factor-1 receptor in regulating microvillus length

**DOI:** 10.1038/s41598-019-49510-5

**Published:** 2019-09-10

**Authors:** Melanie A. Markovic, Patricia L. Brubaker

**Affiliations:** 1 Department of Physiology Rm 3366 Medical Sciences Building, University of Toronto, 1 King’s College Circle, Toronto, ON M5S 1A8 Canada; 2 Department of Medicine Rm 3366 Medical Sciences Building, University of Toronto, 1 King’s College Circle, Toronto, ON M5S 1A8 Canada

**Keywords:** Physiology, Endocrine system and metabolic diseases

## Abstract

Microvilli are tiny projections on the apical end of enterocytes, aiding in the digestion and absorption of nutrients. One of their key features is uniform length, but how this is regulated is poorly understood. Glucagon-like peptide-2 (GLP-2) has been shown to increase microvillus length but, the requirement of its downstream mediator, the intestinal epithelial insulin-like growth factor-1 receptor (IE-IGF-1R), and the microvillus proteins acted upon by GLP-2, remain unknown. Using IE-IGF-1R knockout (KO) mice, treated with either long-acting human (h) (GLY^2^)GLP-2 or vehicle for 11d, it was found that the h(GLY^2^)GLP-2-induced increase in microvillus length required the IE-IGF-1R. Furthermore, IE-IGF-1R KO alone resulted in a significant decrease in microvillus length. Examination of the brush border membrane proteome as well as of whole jejunal mucosa demonstrated that villin was increased with h(GLY^2^)GLP-2 treatment in an IE-IGF-1R-dependent manner. Under both basal conditions and with h(GLY^2^)GLP-2 treatment of the IE-IGF-1R KO mice, changes in villin, IRTKS-1, harmonin, β-actin, and myosin-1a did not explain the decrease in microvillus length, in either the brush border or jejunal mucosa of KO animals. Collectively, these studies define a new role for the IE-IGF-1R within the microvillus, in both the signaling cascade induced by GLP-2, as well as endogenously.

## Introduction

**Figure 1:**
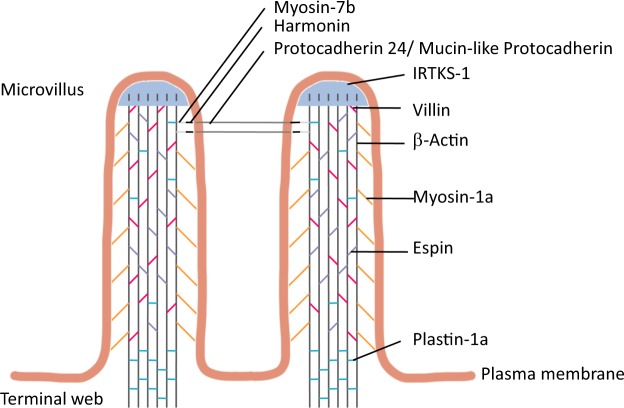
Microvillar proteins and their relative locations within the microvillus.

The major site of nutrient digestion and absorption occurs within the enterocytes of the small intestine. These cells form the majority of the intestinal epithelium, and are highly polarized with the apical end in contact with the lumen of the intestine. Microvilli are tiny, finger-like projections located on the apical surface of enterocytes, and are collectively referred to as the brush border. They increase the absorptive surface area by 9- to 16-fold^1^, and house over 22 digestive enzymes and 53 channels and transporters^2^. In addition, numerous structural proteins are localized to the region to help form and maintain this structure (Fig. 1). Forming the entire microvillus region on the apical surface of the enterocytes requires plasma membrane bending proteins, such as insulin receptor tyrosine kinase substrate-1 (IRTKS-1)^3,4^. Each microvillus has a core made from numerous F-actin filaments, which are bundled together by the actin-bundling proteins: villin, espin, and plastin-1a^5–8^. Myosin-1a, ezrin, and other proteins then tether the actin bundles to both the cytoskeleton and the plasma membrane^9–12^. Finally, intermicrovillar links connect adjacent microvilli, involving several proteins, including harmonin^13^.

A key characteristic of the microvillus is uniform length. Actin monomers are added at the tips and removed distally, by polymerizing and depolymerizing proteins, respectively. Alterations to the addition and/or removal of actin monomers can therefore lead to changes in microvillus length. Length can also be modified by changing the expression of key structural proteins, such as the actin-bundling proteins and myosin-1a^8,9,14^. It has also been shown that the presence of intermicrovillar links is important for maintaining uniform length^13^. However, it is not well understood how these changes in brush border proteins are controlled.

Glucagon-like peptide-2 (GLP-2) is an intestinotrophic hormone, secreted by enteroendocrine L cells of the intestinal epithelium. It has been shown to increase epithelial proliferation, inhibit apoptosis, enhance barrier function, and increase digestion, absorption, and blood flow^15–20^. Interestingly, GLP-2 secretion and microvillus length are both reduced in response to fasting and increase following re-feeding^21,22^. Furthermore, chronic administration of a degradation-resistant GLP-2 receptor agonist, human (h) (GLY^2^)GLP-2, to mice increases microvillus length, to 2-fold greater than that of vehicle treated controls^23^. The GLP-2 receptor is localized on enteroendocrine cells, enteric neurons, and intestinal subepithelial myofibroblasts (ISEMFs), a syncytium of cells located underneath the epithelium^18,24–26^. Since the GLP-2 receptor is not expressed by enterocytes, the effects of GLP-2 on these cells must be induced via downstream mediators. Consistent with this hypothesis, it has been demonstrated that GLP-2 binding to its receptor on the ISEMFs increases the expression and secretion of insulin-like growth factor-1 (IGF-1)^27,28^. Furthermore, GLP-2-induced increases in proliferation and barrier function require IGF-1 and the intestinal epithelial IGF-1 receptor (IE-IGF-1R)^17,27,29–33^. However, it remains unknown as to whether this signaling pathway is also required for GLP-2-induced increases in microvillus length, and, if so, what proteins are involved in these actions.

Therefore, the aim of this study was to determine whether the IE-IGF-1R is required for the increase in microvillus length following GLP-2 treatment, and which brush border proteins are acted upon by GLP-2 to cause the enhancement in microvillus length.

## Results

### Microvillus length depends on the IE-IGF-1R

**Figure 2:**
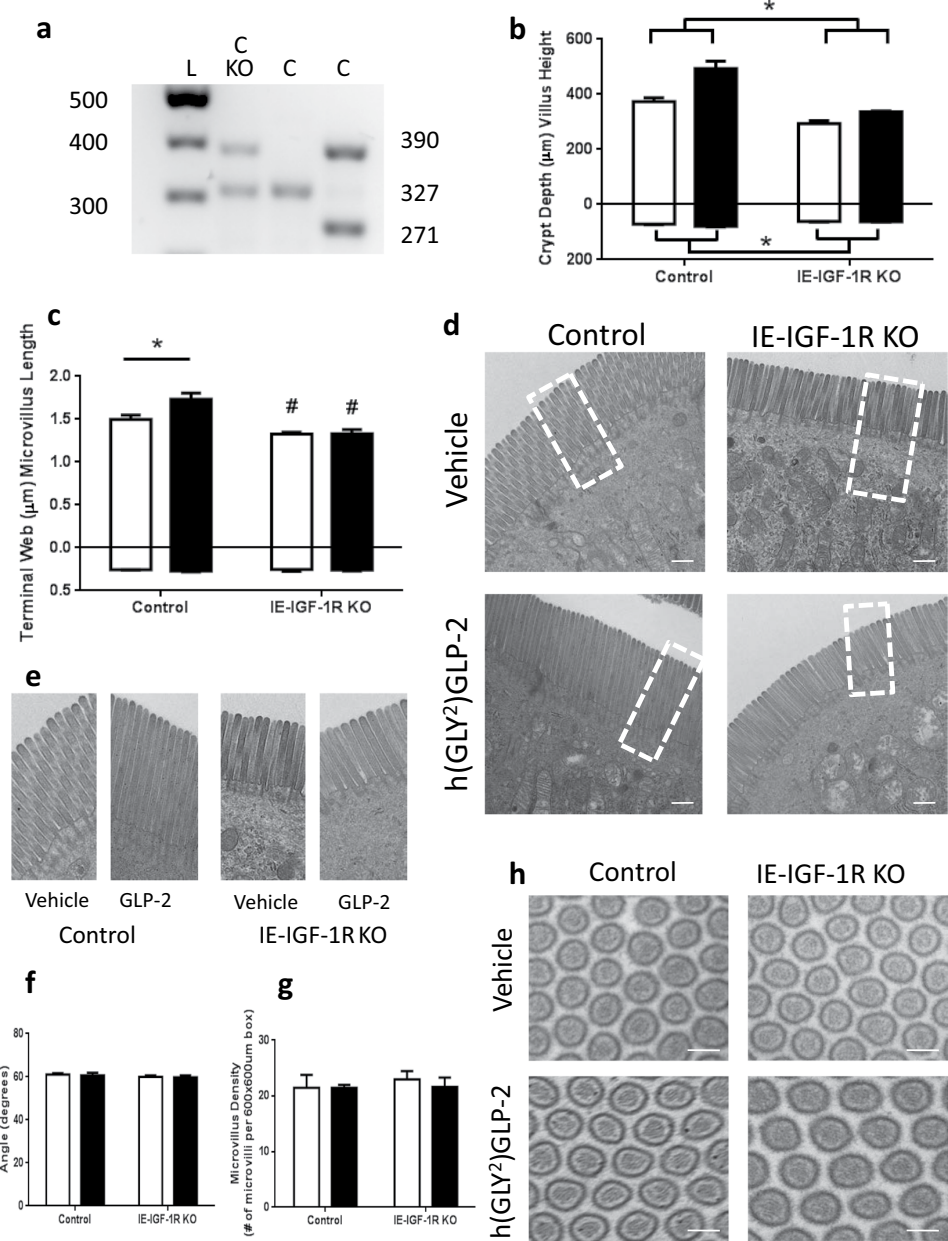
The IE-IGF-1R is required for the GLP-2-induced increase in microvillus length, in addition to regulating length endogenously. (**a**) IE-IGF-1R KO was induced by administration of tamoxifen to mice expressing both the villin-CreER^T2+/0^ (390 bp) and the Igf1r^flox/flox^ (327 bp) genes. Control mice expressed either: villin-CreER^T2+/0^ and the wildtype Igf1r (271 bp) genes; Igf1r^flox/flox^; or both the villin-CreER^T2+/0^ and the Igf1r^flox/flox^ (in the absence of tamoxifen). (**b**) After induction of IE-IGF-1R KO, followed by h(GLY^2^)GLP-2 (closed bars) or vehicle (open bars) treatment for 11d, the lack of a tropic response of IE-IGF-1R KO mice to GLP-2, as compared to control animals, was validated by assessing crypt-villus height (n = 7–11). Microvillus length (**c**–**e**) and packing angle (**f**–**h**) were measured using TEM (representative images are shown in (**d**,**h**), and a magnified view of the boxes marked in (**d**) is shown in (**e**)). n = 5 for microvillus length; microvilli in 28 cells from 5 different villi were measured per mouse. n = 3 for packing angle; a minimum of 40 angles were taken per mouse. n = 3 for microvillus density in a 600 × 600 µm box. *P < 0.05 for the difference between treatment groups, ^#^P < 0.05 for the difference from controls. Scale bar is 100 µm.

Knockout (KO) of the IE-IGF-1R was conditionally induced in mice expressing villin-CreER^T2+/0^; Igf1r^fl^°^x/flox^ (Fig. 2a), and was functionally validated by h(GLY^2^)GLP-2 treatment. Hence, genetic (CreER^T2+/0^, Igf1r^flox/flox^, and CreER^T2+/0^; Igf1r^fl^°^x/flox^) and chemical (ethanol/sunflower oil, and tamoxifen)-treated control animals demonstrated 0.35- and 0.11-fold increases in intestinal villus height and crypt depth, respectively, after 11d of treatment with h(GLY^2^)GLP-2 (p < 0.05; Fig. 2b), whereas IE-IGF-1R KO mice failed to show significant increases in either parameter in response to h(GLY^2^)GLP-2 treatment. Microvillus length was also found to increase to 1.3-fold with h(GLY^2^)GLP-2 treatment in control mice (p < 0.05; Fig. 2c–e). This increase required the IE-IGF-1R, as KO mice administered h(GLY^2^)GLP-2 demonstrated no change in microvillus length. Furthermore, the IE-IGF-1R KO mice, regardless of GLP-2 treatment, exhibited significantly shorter microvilli as compared to vehicle-treated control animals (p < 0.05). To determine whether IE-IGF-1RKO also affected other aspects of MV structure, microvillus density was measured. However, all groups showed the ideal packing angle^9^ of 60°, with no differences in microvillus density (Fig. 2f–h).

### Differential proteomic expression in isolated brush border membranes

**Figure 3:**
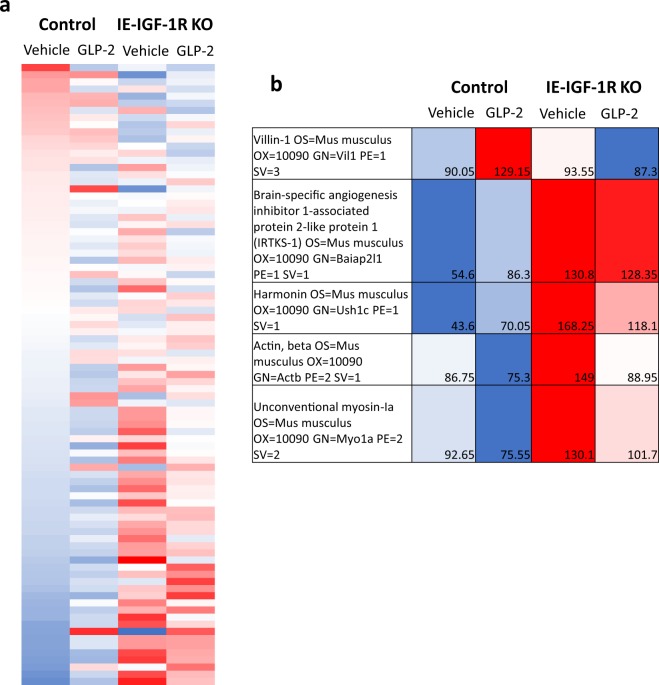
Mass spectrometry on isolated BBMs demonstrated proteins with differential expression, depending on GLP-2 treatment and the presence of the IE-IGF-1R. Control and IE-IGF-1R KO mice were treated with either vehicle or h(GLY^2^)GLP-2 for 11d, followed by brush border isolation from the small intestine. Using Mouse Mines, proteins were assessed for significant gene ontology enrichment for “cellular components”. (**a**) The “actin filament” category was then used to specifically examine structural proteins within the brush border. (**b**) Of these 71 proteins, 5 were selected based on different expression patterns and their known roles in the microvillus.

To understand the changes underlying the alterations in microvillus length, both with GLP-2 treatment and in the IE-IGF-1R KO mice, brush border membranes (BBM) were isolated from control and KO, vehicle- and h(GLY^2^)GLP-2-treated mice. Western blot demonstrated an 11.6-fold enrichment in the brush border protein, villin, as compared to the cytoplasmic protein, GAPDH (preliminary data, not shown). Mass spectrometry analysis identified over 1,200 proteins in the samples (Supplemental Table 1). Mouse Mines (www.mousemines.org) was used to identify the presence of ontologies within the category of “cellular component”, including “cytoskeleton”, “mitochondrial”, “nuclear”, “organelle”, “membrane” and “microvillus”. However, further analysis focused on the “actin filament” gene ontology (GO) category, which was significantly enriched (p < 0.05) and included a total of 71 known proteins, including those that are found in the microvillus. Protein expression levels were then determined for each group of animals relative to the vehicle-treated control mice (Fig. 3a). Marked differences in expression patterns were observed between vehicle- and h(GLY^2^)GLP-2-treated control and IE-IGF-1R KO mice, as well as between control and KO animals for each treatment group. To better understand these differential expression patterns, BBM structural proteins were categorized according to: 1) effects of GLP-2 that required the IE-IGF-1R; and 2) effects of the loss of the IE-IGF-1R alone. Select structural proteins, chosen for their roles in regulating microvillus length, were then analyzed in greater detail.

### Villin

**Figure 4:**
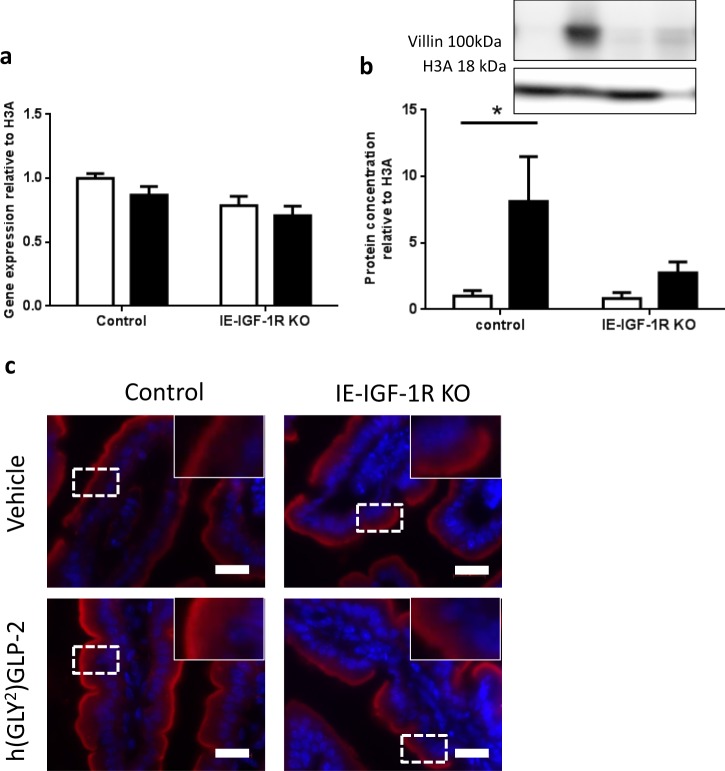
Villin protein expression is increased with GLP-2, in an IE-IGF-1R-dependent manner. Control and KO mice were treated with either vehicle (open bars) or h(GLY^2^)GLP-2 (closed bars) for 11 d, followed by jejunal mucosal scrapes for either (**a**) q-RT-PCR or (**b**) western blot analysis; representative blots are shown, with full length blots in Supplemental Fig. 1 (n = 7–15 for PCR; n = 4–10 for western blot). (**c**) Jejunal sections were fixed in PFA and embedded in OCT for immunofluorescent staining; insets show magnified views of boxed areas. Red is villin, blue is DAPI. Scale bar is 20 µm (n = 4). *P < 0.05.

In the isolated BBM, VILLIN demonstrated a significant correlation (R^2^ = 0.97; p < 0.05) for effects of h(GLY^2^)GLP-2 that require the IE-IGF-1R (category 1; Fig. 3b). VILLIN expression was increased to 1.43-fold in h(GLY^2^)GLP-2 treated control mice, with no change seen in h(GLY^2^)GLP-2 treated IE-IGF-1R KO animals. To further understand villin expression changes, the jejunal mucosa was analyzed in greater detail. Although *Villin* mRNA levels showed no difference between groups (Fig. 4a), changes in VILLIN protein levels, as determined by immunoblot, paralleled the BBM findings, with an 8.13-fold increase in the h(GLY^2^)GLP-2 treated control mice (p < 0.05; Fig. 4b). Immunofluorescence analysis of jejunal sections supported these findings (Fig. 4c), as control mice given h(GLY^2^)GLP-2 showed suggested increases in the intensity of VILLIN staining at the brush border. Furthermore, the GLP-2-induced increase in VILLIN required the IE-IGF-1R, as KO mice failed to show significant increases in protein expression as well as staining intensity.

### IRTKS-1

**Figure 5:**
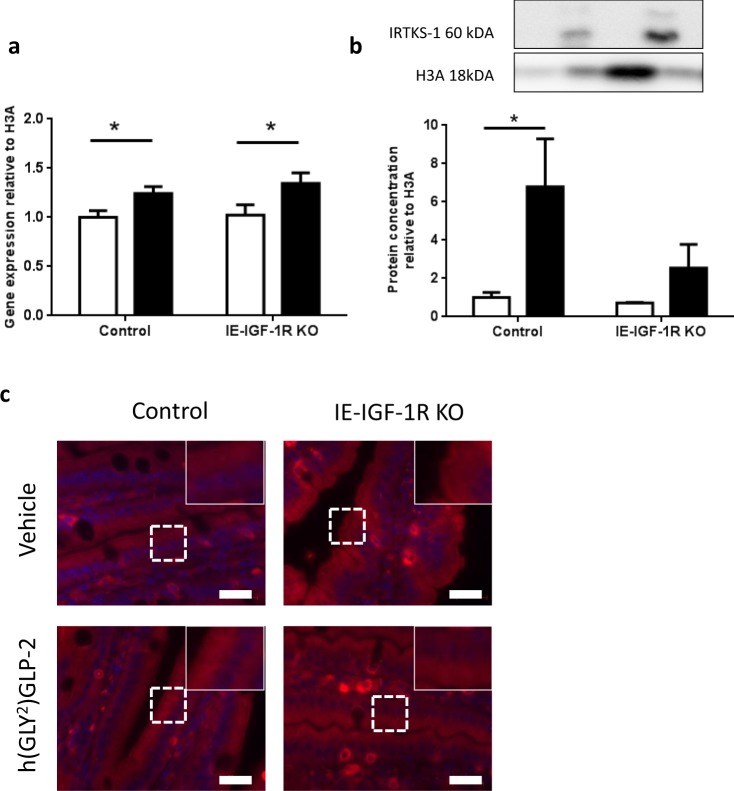
In the mucosa, IRTKS-1 protein is increased with GLP-2 treatment in an IE-IGF-1R-dependent manner. Control and IE-IGF-1R KO mice were treated with either vehicle (open bars) or h(GLY^2^)GLP-2 (closed bars) for 11 d, followed by jejunal mucosal scrapes for either (**a**) q-RT-PCR or (**b**) western blot analysis; representative blots are shown, with full length blots in Supplemental Fig. 2 (n = 8–14 for PCR; n = 2–6 for western blot). (**c**) Jejunal sections were paraffin embedded for immunofluorescent staining; insets show magnified views of boxed areas. Red is IRTKS-1, blue is DAPI. Scale bar is 20 µm (n = 4). *P < 0.05.

IRTKS-1 levels in the BBM demonstrated a significant correlation (R^2^ = 0.93; p < 0.05) for effects of the IE-IGF-1R KO alone, being higher in both the vehicle (2.39-fold) and h(GLY^2^)GLP-2 treated (1.38-fold) KO mice, as compared to the control animals (category 2; Fig. 3b). Interestingly, although mRNA transcript levels were increased by GLP-2 treatment in both groups of animals (p < 0.05; Fig. 5a), the increase in IRTKS-1 in the IE-IGF-1R KO BBM was not observed by western blot of the jejunal mucosa. Instead, there was a 6.80-fold increase in IRTKS-1 levels in the h(GLY^2^)GLP-2 treated control mice, and a smaller, non-significant increase in the KO animals (Fig. 5b). These changes were not observable under immunofluorescence, with the intensity and localization remaining unchanged (Fig. 5c).

### Harmonin

**Figure 6:**
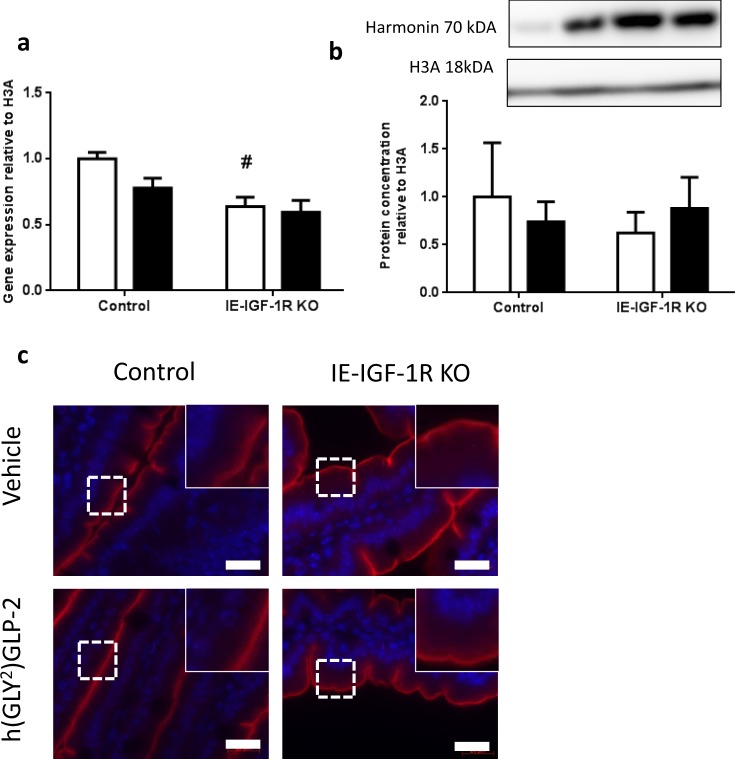
No change in harmonin expression within the jejunal mucosa. Control and IE-IGF-1R KO mice were treated with either vehicle (open bars) or h(GLY^2^)GLP-2 (closed bars) for 11 d, followed by jejunal mucosal scrapes for either (**a**) q-RT-PCR or (**b**) western blot analysis; representative blots are shown, with full length blots in Supplemental Fig. 3 (n = 8–15 for PCR; n = 7–9 for western blot). (**c**) Jejunal sections were paraffin embedded for immunofluorescent staining; insets show magnified views of boxed areas. Red is harmonin, blue is DAPI. Scale bar is 20 µm (n = 4). ^#^P < 0.05 compared to vehicle control.

In the BBM, HARMONIN also had a significant correlation (R^2^ = 0.95; p < 0.05) for effects of the IE-IGF-1R KO alone, with 3.85-fold and 1.69-fold increases in expression in vehicle and h(GLY^2^)GLP-2 treated IE-IGF-1R KO mice, respectively (category 2; Fig. 3b). However, harmonin in the entire jejunal mucosa showed different results. *Harmonin* transcript levels were significantly decreased in the IE-IGF-1R KO vehicle mice, compared to those in the controls (p < 0.05; Fig. 6a), whereas the mucosa showed no change in HARMONIN expression in response to either h(GLY^2^)GLP-2 treatment or the KO (Fig. 6b). Furthermore, this trend was not apparent by harmonin immunostaining of the jejunum, as relative intensities and localization appeared similar between groups, with localization predominantly in the brush border (Fig. 6c).

### β-actin

**Figure 7:**
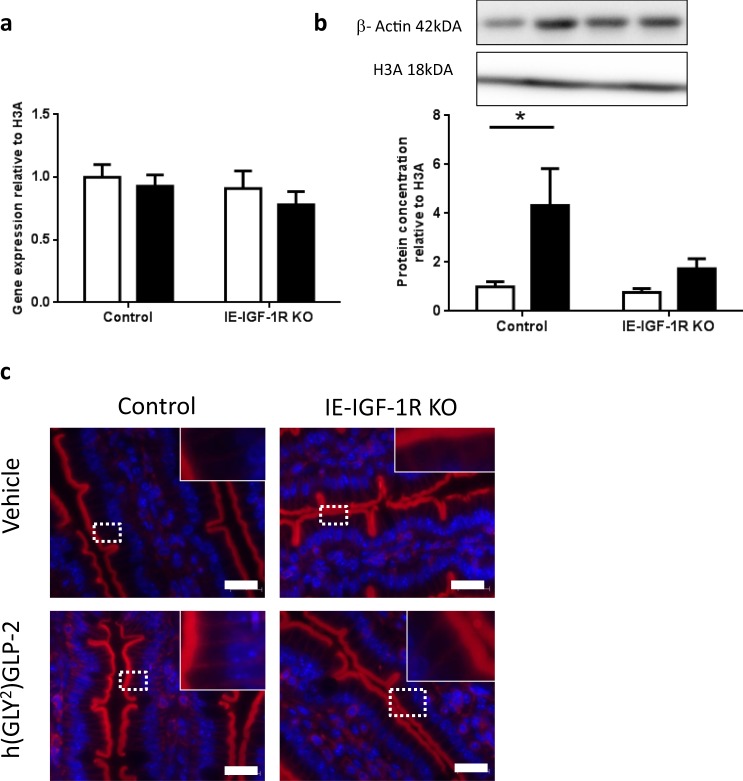
β-actin protein increases with GLP-2, in an IE-IGF-1R-dependant manner. Control and IE-IGF-1R KO mice were treated with either vehicle (open bars) or h(GLY^2^)GLP-2 (closed bars) for 11 d, followed by jejunal mucosal scrapes for either (**a**) q-RT-PCR or (**b**) western blot analysis; representative blots are shown, with full length blots in Supplemental Fig. 4 (n = 8–14 for PCR; n = 8–10 for western blot). (**c**) Jejunal sections were paraffin embedded for immunofluorescent staining; insets show magnified views of boxed areas. Red is β-actin, blue is DAPI. Scale bar is 20 µm (n = 4). *P < 0.05.

In the BBM, β-ACTIN had a significant correlation (R^2^ = 0.95; p < 0.05) for effects of the IE-IGF-1R KO alone (category 2; Fig. 3b). However, only the vehicle treated KO mice had higher β-ACTIN levels, with a 1.75-fold increase as compared to h(GLY^2^)GLP-2 treated KO mice, which demonstrated only a non-significant 1.18-fold increase. No changes in *β-actin* transcript levels were detected in the jejunal mucosa (Fig. 7a), while examination of protein levels in these tissue samples showed different results as compared to the isolated BBM, such that β-ACTIN showed a significant 4.3-fold increase in the jejunal mucosal of h(GLY^2^)GLP-2 treated control mice (p < 0.05; Fig. 7b). This finding was supported by immunostaining, where observable increases in fluorescent intensity were observed in h(GLY^2^)GLP-2 control mice, in both the brush border and basolaterally (Fig. 7c). This increase in β-ACTIN in the intestinal mucosa required the IE-IGF-1R, as the KO mice treated with h(GLY^2^)GLP-2 failed to demonstrate increases in protein expression, or in the intensity of the immunostaining in the brush border and basolaterally.

### Myosin-1a

**Figure 8:**
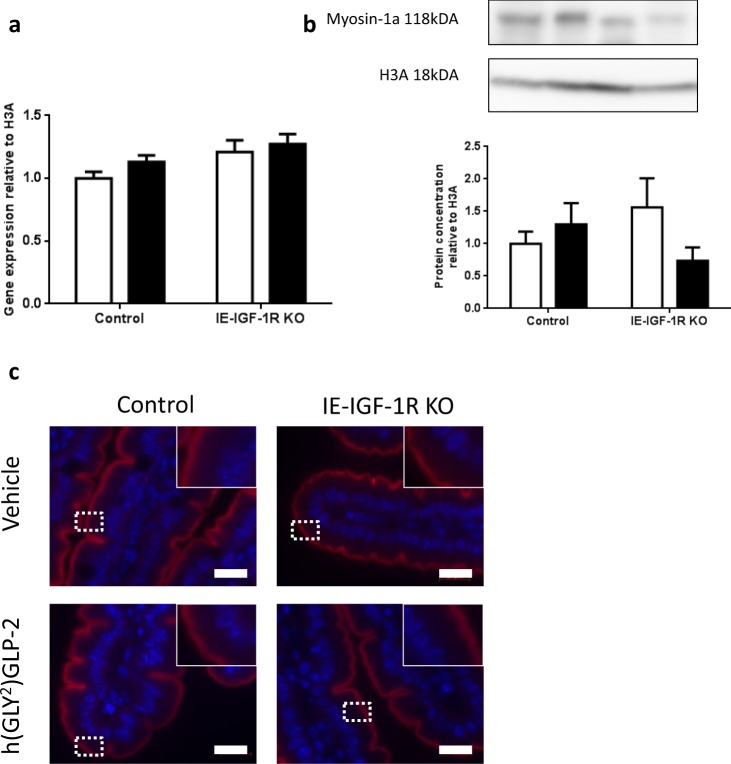
Myosin-1a remains unchanged with both GLP-2 treatment and IE-IGF-1R KO. Control and IE-IGF-1R KO mice were treated with either vehicle (open bars) or h(GLY^2^)GLP-2 (closed bars) for 11 d, followed by jejunal mucosal scrapes for either (**a**) q-RT-PCR or (**b**) western blot analysis; representative blots are shown, with full length blots in Supplemental Fig. 5 (n = 8–14 for PCR; n = 4–9 for western blot). (**c**) Jejunal sections were paraffin embedded for immunofluorescent staining; insets show magnified views of boxed areas. Red is myosin-1a, blue is DAPI. Scale bar is 20 µm (n = 4).

Finally, in the BBM, MYO-1A did not show a significant correlation with either GLP-2 treatment or IE-IGF-1R KO (Fig. 3b). Analyses of the jejunal mucosa also failed to demonstrate any changes in jejunal mucosal expression of myo-1a at either the mRNA or protein levels (Fig. 8).

## Discussion

Microvilli display uniform length, which is of functional importance^1^. Proteins within the BB region have therefore been studied to better understand their influence on microvillus length; however, the factors that regulate these proteins are poorly understood. The results of the present study indicate that the expression of several proteins with key roles in brush border structure is differentially regulated by the intestinotrophic hormone, GLP-2, as well as by one of its downstream signaling mediators, the IE-IGF-1R.

The BBM proteome has been isolated previously, and was reported to express approximately 650 identified proteins^2^. Unexpectedly, the isolation conducted herein contained over 1,200 proteins. Although the isolation protocols were identical, one key difference that may account for such a drastic difference in the number of identified proteins was the mass spectrometry instrument. The Orbitrap Fusion Lumos, used in the current study, has a faster scanning time and is more sensitive, as compared to the Thermo Scientific LTQ linear ion-trap utilized previously^2^, and therefore represents a technological advance given the almost ten-year time gap between the two proteomes. This may allow for the identification of previously unidentified brush border proteins, leading to enhanced understanding of the development and maintenance of this critical intestinal epithelial cell compartment.

The BBM isolation reported in this paper was validated in two ways. First, through western blot, probing for the ratio of VILLIN to GAPDH in both BBM and jejunal mucosa samples. VILLIN is a protein that, within the enterocytes, is only found in the brush border^34^; thus, the ratio of VILLIN:GAPDH should be, and was found to be, higher in the BBM isolate. Another form of validation was conducted by analysis of the proteome using Mouse Mines, with the results showing protein categories as previously reported^2^, and significant up regulation of specific GO terms, under “cellular component” ontologies, that one would expect to find in the brush border, such as “brush border”, “microvillus”, “cluster of actin-based projections”, and “actin filament bundles”.

h(GLY^2^)GLP-2 has been previously reported to increase microvillus length^23^, but how this is achieved is poorly understood. Through the use of the IE-IGF-1R KO mice, we have now shown that the increase in microvillus length with h(GLY^2^)GLP-2 treatment requires the IE-IGF-1R. One key microvillus protein that was regulated by the GLP-2 - IE-IGF-1R pathway was villin, an actin-bundling protein^35–37^, with a well-established role in epithelial wound repair^38^. Although it’s deletion is not sufficient to abolish the formation of microvilli^5^, villin over-expression has been shown to induce the expression of membrane projections in cells that do not naturally do so^39^. In the present study, the protein but not the transcript levels of villin were increased with h(GLY^2^)GLP-2 treatment, in an IE-IGF-1R-dependent manner. Decreases in VILLIN levels have been reported previously in patients with Crohn’s Disease, while mRNA levels also remained unchanged^40,41^. These findings suggest possible translational regulation of villin, as opposed to transcriptional modulation. Furthermore, with links to decreased IGF-1 levels in Crohn’s disease patients^42^, in parallel with the reductions in villin, this suggests a new mechanism by which h(GLY^2^)GLP-2 may attenuate Crohn’s disease^43^ and/or other inflammatory enteropathologies. Future studies involving GLP-2 administration to intestinal epithelial villin-deficient animals, with and without induced enteritis, may prove useful to further interrogate this pathway.

Another interesting result was the lack of increase in the core-forming protein, β-actin, in the BBM of control mice treated with h(GLY^2^)GLP-2. With an increase in microvillus length, it would follow that there should be an increase in β-actin within the BBM, as this protein forms the core. However, the overall increase in microvillus length, although significant, was only 0.2 µm and, thus, may have been too small to detect an increase in β-actin expression. It has been shown that the ratio of β-actin to villin expression is 1:4^44^; thus, increases in villin expression are more likely to be detected as compared to β-actin, within the BBM itself. However, when the entire jejunal mucosa was analyzed, there was a significant increase in β-actin expression with h(GLY^2^)GLP-2 treatment, which required the IE-IGF-1R. This result is also consistent with the immunostaining, which showed an increase in the intensity of the basolateral staining. Thus, the GLP-2 - IE-IGF-1R pathway is also important for regulating the expression of β-actin in the jejunal mucosa.

Unexpectedly, the IE-IGF-1R KO mice presented with shorter microvilli. The IE-IGF-1R has not been reported to play a role in regulating microvillus length, with IGF-1 and its receptor better known for their roles in whole body growth^45,46^, metabolism^47^, and cancer^48^. Focusing on the intestine, IGF-1 and its receptor are major determinants in colorectal cancer^49^ and may be decreased in some patients with Crohn’s disease^42^. IGF-1 signaling has also been shown to mediate the intestinotrophic effects of h(GLY^2^)GLP-2^17,29,31–33^, as well as to stimulate Na^+^/K^+^-ATPase activity and enhance Na^+^-coupled glucose absorption in enterocytes^50^. The present studies therefore define a new role for the IGF-1R in regulating the length of the intestinal brush border.

Decreases in microvillus length in response to BBM protein KO have been reported previously^8,9,11,13,14^, but these models also demonstrate changes in the actin core cytoplasmic projections (rootlets)^8,14^, architecture^9,11,13^, integrity of the plasma membrane^9^, and/or microvillus membrane^11^, hallmark features that were not present in the IE-IGF-1R KO mice. Plastin-1a KO mice have 20% shorter microvilli, but also lack rootlets, which was not observed with IE-IGF-1R KO^8^. Similarly, combinatory KO of villin, espin, and plastin-1a decreases both microvillus and rootlet length^14^. In contrast, KO of desmoplakin, a desmosomal protein, results in microvilli that are shorter but also misshaped, the latter of which was not seen in the IE-IGF-1R KO mice^51^. Harmonin KO animals also display shorter microvilli, in addition to changes in architecture and density that were not observed in the IE-IGF-1R KO^13^. Finally, KO of myo-1a results in longer, less densely packed microvilli, with extrusions of cellular cytoplasm in the intestinal lumen^9^, again, a phenotype that was not noted in the IE-IGF-1R KO model. Collectively, these findings indicate a unique role for the IGF-1R in regulating proteins that contribute to microvillus length without affecting microvillus density or structure.

IRTKS-1 is known to play an important role in membrane bending and microvillus formation^3,52^ and was recently reported to increase microvillus length during enterocyte differentiation^4^. IRTKS-1 may be regulated by the IE-IGF-1R, as the isolated BBM showed a significant correlation wherein the KO mice had higher expression levels than controls. What is still unclear is whether the IE-IGF-1R negatively regulates the expression of IRTKS-1, or if this protein is increased in the KO mice in compensation for the decrease in microvillus length. The jejunal mucosa analysis indicates the latter of the two possibilities. IRTKS-1 is not exclusively located in the BBM so, upon measuring the protein levels in the mucosa, they remained unchanged in the vehicle KO mice compared to vehicle controls, showing the IE-IGF-1R does not regulate overall expression of IRTKS-1. Furthermore, although h(GLY^2^)GLP-2 increased IRTKS-1 in the mucosa, with a smaller concentration of IRTKS-1 located in the BBM, the effects of GLP-2 were undetectable in this compartment. Instead, the BBM proteome showed a compensatory action of the enterocyte to elongate its microvillus. Interestingly, espin-8 expression in the BBM also showed increases in the IE-IGF-1R KO, as compared to the controls (data shown in Supplemental Table 1). Espin-8 has been reported to play a role in the effects of IRTKS-1 on the brush border^4^. The increase in espin-8 therefore supports a role for the increased IRTKS-1 in the KO mice as a compensatory attempt to enhance microvillus length.

The change in harmonin expression with the BBM may also indicate another compensatory change in the IE-IGF-1R KO to increase microvillus length. Harmonin levels were positively correlated with the effects of the KO alone, such that the KO mice, regardless of treatment, had higher levels of harmonin as compared to the control animals. Harmonin is known to be expressed primarily in the BBM^13^, as confirmed by the pattern of immunostaining, so it was surprising that the jejunal mucosa did not mirror this result. However, the differential sensitivities of the two methods must be considered and, therefore, their ability to detect changes in a protein which is expressed in relatively small amounts.

Myosin-1a is a protein that is exclusive to the brush border^53^ and, accordingly, was found to be expressed in both the BBM and jejunal mucosa, with predominant localization to the brush border. However, myosin-1a is known to be important for membrane-actin cytoskeletal adhesion^9^ and myosin-1a KO mice present with membrane extrusions and decreased microvillus density, hallmark features that were not seen here in response to either GLP-2 treatment or IGF-1R KO.

Although GLP-2 treatment is known to increase intestinal digestive and absorptive capacity^16,19,54,55^, KO of the GLP-2R reduces amino acid absorption but does not impact whole body growth^56^. It still remains unclear as to whether there are any functional consequences following IE-IGF-1R KO except for an inability to increase proliferation^29^ or barrier function^17^ in response to GLP-2 administration. However, no changes in body weight have been reported for the IE-IGF-1R KO mice^17,29^, as also found in the present study (data not shown). Notwithstanding, these studies have all been conducted for only two-weeks following induction of the KO which, given the relatively short time in additional to the small decrease in microvillus length, may not be sufficient to detect a robust change in body weight. Indeed, this is consistent with other KO models, wherein key brush border proteins are absent but no overall physiological effect is seen^5,8,9,13,14,57^. Consistent with this possibility, myo-1a, which was unaffected by IE-IGF-1R KO, has been shown to also be important in membrane trafficking within the microvillus^9^, and sucrase isomaltase and alkaline phosphatase were detected at normal levels in the BBM from the IE-IGF-1R KO (Supplemental Table 1), suggestive of normal trafficking.

Finally, one limitation of this study is the use of whole small intestine to prepare the brush border proteome, as compared to the molecular analyses of jejunal mucosa and full-thickness cross-sections. As the GLP-2 receptor is expressed at highest levels in the jejunum^24^, many studies have focused on this region of the gut^17,29,54,55^. However, as a consequence, changes in the proteome may also reflect differences in duodenal and/or ileal microvillar proteins that are not occurring in the jejunum. Further studies will be required to determine the possibility of site-specific differences in the regulation of microvillus length.

In conclusion, the results of the present study demonstrate not only that the IE-IGF-1R is essential for the GLP-2 induced increase in microvillus length, but that it also plays a key role in the maintenance of basal microvillus length under non-stimulated conditions. Although changes in several key microvillus proteins were demonstrated in response to experimental manipulation of these systems, the complex interplay of these proteins and the downstream mechanism(s) by which they regulate microvillus length remain to be fully elucidated.

## Methods

### Animals

As previously described^17,29^, IE-IGF-1R KO mice were generated by crossing villin-CreER^t2+/0^ and Igf1r^flox/flox^ mice, both on a C57BL/6 background^58,59^. The villin-CreER^T2+/0^; Igf1r^flox/0^ offspring were then backcrossed to Igf1r^flox/flox^ mice, and the KO was induced by ip injection of Tamoxifen (100 µl of 10 mg/mL in ethanol and sunflower oil) for 5 d^17,29^. Mice were genotyped by detection of the wild-type Igf-1r and Igf-1r floxed alleles (5′-ATCTTGGAGTGGTTGGGTCTGTTT-3′ and 5′-ATGAATGCTGGTGAGGGTTGTCTT-3′), and the Cre allele (5′-CCTGGAAAATGCTTCTGTCCG-3′ and 5′-CAGGGTGTTATAAGCAATCCCC-3′) (Fig. 2a). Control mice included: Igf1r^flox/flox^ ± tamoxifen, villin-CreER^t2+/0^ ± tamoxifen, and villin-CreER^T2+/0^; Igf1r^flox/flox^ - tamoxifen. KO mice were villin-CreER^T2+/0^; Igf1r^flox/flox^ + tamoxifen. Sex (male and female)- and age (8–15 wk)-matched littermates were used in all experiments, with the exception of the villin-CreER^t2+/0^ animals which were colony- but not litter-matched due to the breeding paradigm. Animals were housed in an animal facility with a 14-hr light, 10-hr dark cycle at the University of Toronto. All studies were approved by the University of Toronto Animal Care Committee. All methods were performed in accordance with the guidelines and regulations of the Canadian Council on Animal Care.

Following induction, 0.1 μg/g h(GLY^2^)GLP-2 (American Peptide Company, Sunnyvale, CA) or vehicle (PBS) was injected sc q24hr for 11 d, with the final treatment given 3 hr prior to euthanasia^17,29,32,33^. The small intestine was collected and flushed with cold PBS. Multiple 2 cm mid-jejunal sections were then fixed in 10% formalin for 24 hr for paraffin embedding and sectioning (University Health Network, Toronto, ON), fixed in paraformaldehyde for 24 hr for frozen sectioning, fixed in 2.5% glutaraldehyde in Sorensen buffer for transmission electron microscopy (TEM), or frozen on dry ice and stored at −80 °C for mRNA and protein analysis; alternatively, the entire small intestine was collected for brush border membrane (BBM) isolation.

### BBM isolation

As previously described^2^, small intestines from 20 mice were dissected, flushed with cold saline, and cut open to expose the luminal surface. They were then cut into small pieces and combined in sucrose dissociation solution (200 mM sucrose, 0.02% Na-azide, 12 mM EDTA-K, 18.9 mM KH_2_PO_4_, and 78 mM Na_2_HPO_4_; pH 7.2), with stirring for 30 min at 4 °C. After filtering through cheesecloth, the liquid was spun at 200 g for 8 min, and then washed twice with sucrose dissociation solution, at 200 g for 8 min. The cell pellet was re-suspended in cold homogenization buffer (10 mM imidazole, 4 mM EDTA-K, 1 mM EGTA-K, 0.02% Na-azide, 1 mM DTT, and 1 mM Pefabloc-SC; pH 7.2) and homogenized in a blender (Hamilton Beach, ‘smoothie’ setting) for 4 × 15 sec. After another centrifugation (500 g, 8 min), the pellets were washed twice in homogenization buffer with 0.5 mM Pefabloc-SC (500 g, 8 min), and then washed twice in Solution A (75 mM KCl, 10 mM Imidazole, 1 mM EGTA, 5 mM MgCl_2_, and 0.02% Na-azide; pH 7.2). After resuspension of the pellet in Solution A, a 60% sucrose solution was added to make a final concentration of 50%. A 40% sucrose solution was layered on top, and the samples were spun at 146,900 g for 1.5 hr (Beckman Optima XPN 80, SW70Ti rotor). The interface between the two gradients, containing the isolated BBM was then removed and washed twice with solution A (500 g, 8 min), and stored at −80 °C. The isolated protein was quantified by BCA assay (Pierce BCA Assay, Thermo Scientific), and underwent western blot validation.

Mass spectrometry was performed by the SPARC BioCentre (The Hospital for Sick Children, Toronto, ON, Canada), using the Thermo Scientific Orbitrap Fusion-LumosTribid Mass Spectrometer (ThermoFisher, San Jose, CA) outfitted with a nanospray source and EASY-nLC 1200 nano-LC system (ThermoFisher, San Jose, CA) and equipped with ETD mode. Lyophilized peptide mixtures were dissolved in 0.1% formic acid and loaded onto a 75 μm × 50 cm PepMax RSLC EASY-Spray column filled with 2 μM C18 beads (ThermoFisher San, Jose CA) at a pressure of 900Bar and a temperature of 60 °C. Peptides were eluted over 240 min at a rate of 250 nL/min using a gradient set up as follows, where Buffer A is 0.1% formic acid and Buffer B is 80% acetonitrile, 0.1% formic Acid, all v/v in HPLC grade water: 0–100 min, 3–25%B; 100–228 min, 25–44%B; 228–230 min, 44–100%B; 230–240 min, 100%B.

Peptides were introduced by nano-electrospray into the Fusion- Lumos mass spectrometer (Thermo-Fisher). Data were acquired using the MultiNotch MS3 acquisition with synchronous precursor selection (SPS) with a cycle time of 5 sec. MS1 acquisition was performed with a scan range of 550 m/z–1800 m/z with resolution set to 120 000, maximum injection time of 50 msec and AGC target set to 4e5. Isolation for MS2 scans was performed in the quadrupole, with an isolation window of 0.7. MS2 scans were done in the linear ion trap with a maximum injection time of 50 msec and a normalized collision energy of 35%. For MS3 scans, HCD was used, with a collision energy of 30% and scans were measured in the orbitrap with a resolution of 50000, a scan range of 100 m/z–500 m/z, an AGC Target of 3e4, and a maximum injection time of 50 msec. The dynamic exclusion was applied using a maximum exclusion list of 500 with an exclusion duration of 20 sec.

### Mouse mines analysis

A total of 1,282 proteins were identified, and run through Mouse Mines (www.mousemines.org), returning a list of 1,223 genes. Parent ontologies, as well as significantly enriched gene ontologies, were determined. The significantly enriched “actin filament” gene ontology was used to narrow down the list to 71 proteins relevant to the brush border region. Protein spectral counts were then normalized to the highest count, giving new values ranging from 0.01–1.0. These normalized values were then analyzed by Pearson correlations against models which showed differential expression between groups. A change was defined as any normalized value which was ≥0.3 compared to other groups, and no change between groups was defined as having a ≤ 0.1 difference in normalized expression, with 0.5 as the baseline.

### Microscopy

Crypt-villus height was measured on hematoxylin/eosin-stained slides, with a minimum of 20 villi and crypts per mouse to make n = 1 (AxioVision software, Carl Zeiss, Canada). For immunofluorescent staining, one mouse from each treatment and each genotype group was represented on each slide. After xylene dewaxing and rehydration for paraffin-embedded slides, or warming up to room temperature for frozen slides, they underwent heat-induced antigen retrieval, using citrate buffer for VILLIN, and Tris-EGTA for MYOSIN-1A, β-ACTIN, HARMONIN and IRTKS-1 (*Baiap2l1*). Tissues were blocked in 10% normal goat serum, and incubated with primary antibodies (Supplemental Table 2) overnight at 4 °C. Secondary antibodies (Supplemental Table 2) were added for 1 hr, in the dark, at room temperature. An AxioPlan epifluorescence microscope (Carl Zeiss, Canada) was used for all imaging, with constant exposure times used for all 4 samples per slide. Z-stacking and deconvolution was conducted using the AxioVision software when required. Negative controls were run by omission of all primary antisera (Supplemental Fig. 6).

For TEM, glutaraldehyde fixed tissues were post-fixed in 2% phosphate-buffered OsO_4_, followed by dehydration in acetone, and embedding and polymerization in Epon-Araldite (Hospital for Sick Children, Toronto, Ontario, Canada). For determining microvillus and terminal web length, a minimum of 4 measurements was taken for each cell for a total of 28 cells from at least 5 different villi per mouse to make n = 1, on a Hitachi H-7000 electron microscope. Microvillus length was determined, using the Hitachi software, by measuring from the distal tip to the plasma membrane, and the terminal web was measured from the plasma membrane to the end of cytoplasmic projection. Packing angle was determined by taking a minimum of 40 measurements of the angle between three adjacent microvillus cross sections, to make n = 1 for each mouse. Microvillus density was determined as the number of intact microvillus cross-sections within a 3.6 × 10^8^ µm^2^ box, for n = 3 mice per group.

### RNA isolation and real-time quantitative reverse-transcription polymerase chain reaction (q-RT-PCR)

Total RNA was isolated from jejunal mucosal scrapes using the RNeasy kit with QiaShredder (Qiagen, Inc, Mississauga, ON, Canada), converted into complementary DNA (5x all-in-one RT Master Mix, abm), and assessed by Taqman Gene Expression Assay (Applied Biosystems, Foster City, CA**)**, using TaqMan primers as shown in Supplemental Table 3). The delta-delta CT method was used to normalize relative mRNA levels^60^.

### Western blot

Jejunal mucosal scrapes were sonicated in RIPA lysis buffer, and protein contained in BBM isolates and mucosal scrapes was quantified by BCA assay (Pierce BCA Assay, Thermo Scientific). Proteins were run on an 8% PAGE gel, transferred onto a PVDF membrane, and incubated with primary antibodies overnight at 4 °C (Supplemental Table 4). Secondary antibodies were incubated for 1 hr, at room temperature (Supplemental Table 4), and visualized using ECL detection reagent (Cell Signaling Technology). Samples were normalized to small intestinal controls where appropriate, and all samples were normalized to vehicle-treated control mice. Each membrane was cut into two pieces by molecular weight ladder (i.e. at ~35–37 kDa), and probed for the protein of interest (i.e. villin, IRTKS-1, harmonin, β-Actin or myo-1a) and the paired internal control (H3A).

### Statistical analysis

All data are expressed as mean + SEM. Two-way ANOVA was performed, followed by Sidak post-hoc test when appropriate. Significance was set at 0.05 for all tests, and all analyses were conducted using GraphPad Prism.

## Supplementary information

Supplementary Figures S1-S6

Supplementary Table S1

## Data Availability

The mass spectrometry dataset analyzed for this study is included in this published article as Supplemental Table 1.
