# Supplementary material for: The roles of glucagon-like peptide-2 and the intestinal epithelial insulin-like growth factor-1 receptor in regulating microvillus length
Source: Sci Rep. 2019 Sep 10;9:13010. doi: 10.1038/s41598-019-49510-5 (PMC6737075; doi:10.1038/s41598-019-49510-5)
Supplement: Supplementary file 1 — Supplementary Figures S1-S6 [file 41598_2019_49510_MOESM1_ESM.pdf]

The roles of glucagon-like peptide-2 and the intestinal epithelial insulin-like growth factor-1 receptor in regulating microvillus length

Melanie A. Markovic and Patricia L. Brubaker

Supplementary Figure Legends

<u>Figure S1</u>: Membrane from one gel was cut and probed for both Villin and H3A. Boxed areas indicate bands shown in manuscript.

<u>Figure S2</u>: Membrane from one gel was cut and probed for both IRTKS and H3A. Boxed areas indicate bands shown in manuscript.

<u>Figure S3</u>: Membrane from one gel was cut and probed for both Harmonin and H3A. Boxed areas indicate bands shown in manuscript.

<u>Figure S4</u>: Membrane from one gel was cut and probed for both β-Actin and H3A. Boxed areas indicate bands shown in manuscript.

<u>Figure S5</u>: Membrane from one gel was cut and probed for both Myo-1a and H3A. Boxed areas indicate bands shown in manuscript.

<u>Figure S6</u>: Representative negative controls images for immunostaining shown in manuscript.

Villin

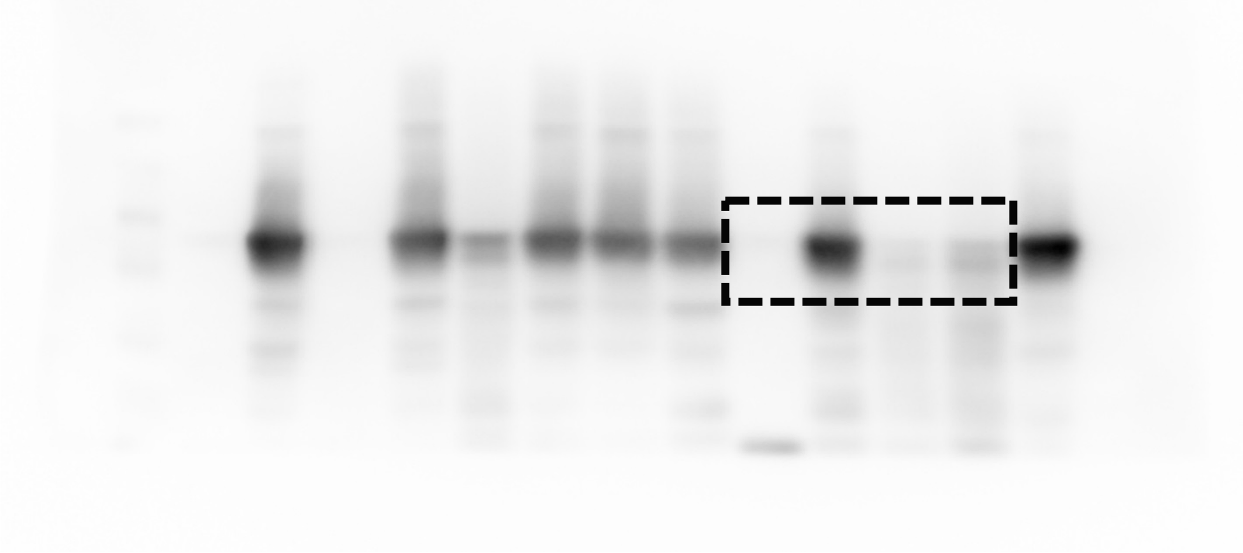

H3A

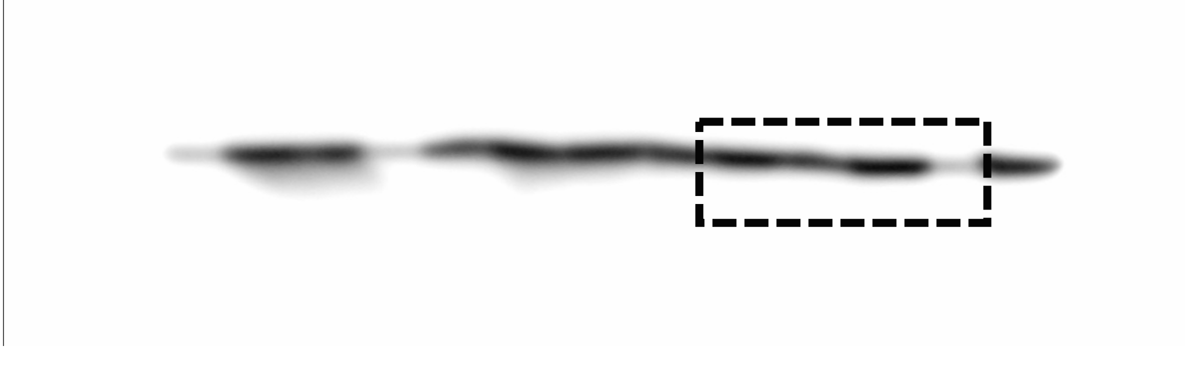

Supplemental Fig. 1

IRTKS-1

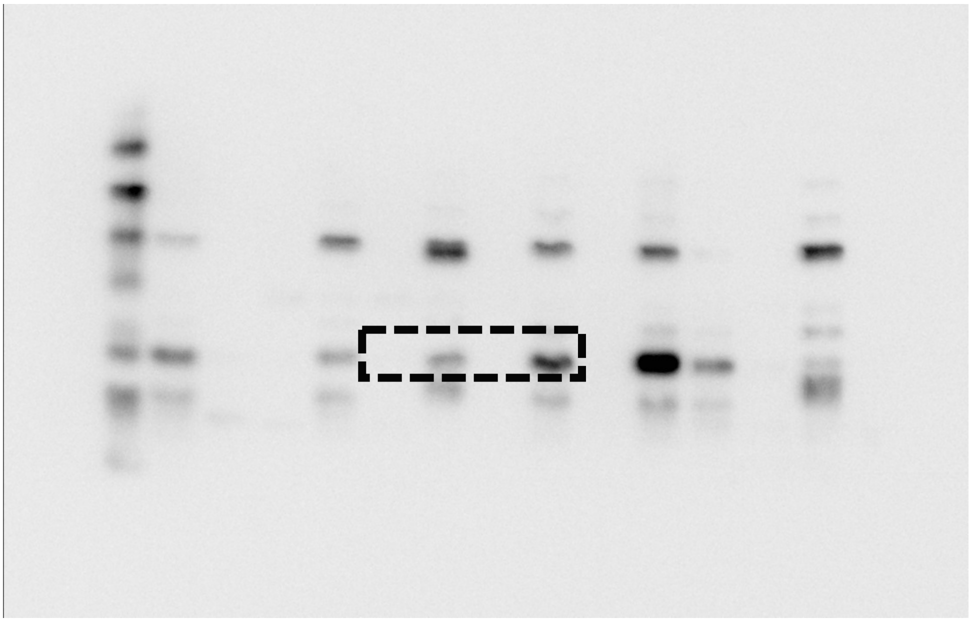

H3A

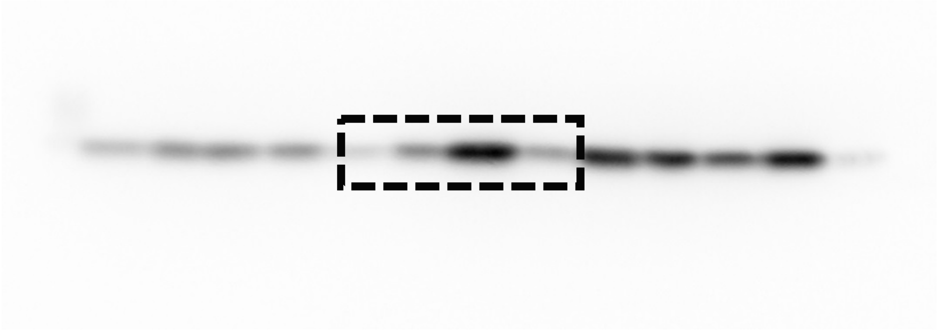

Supplemental Fig. 2

Harmonin

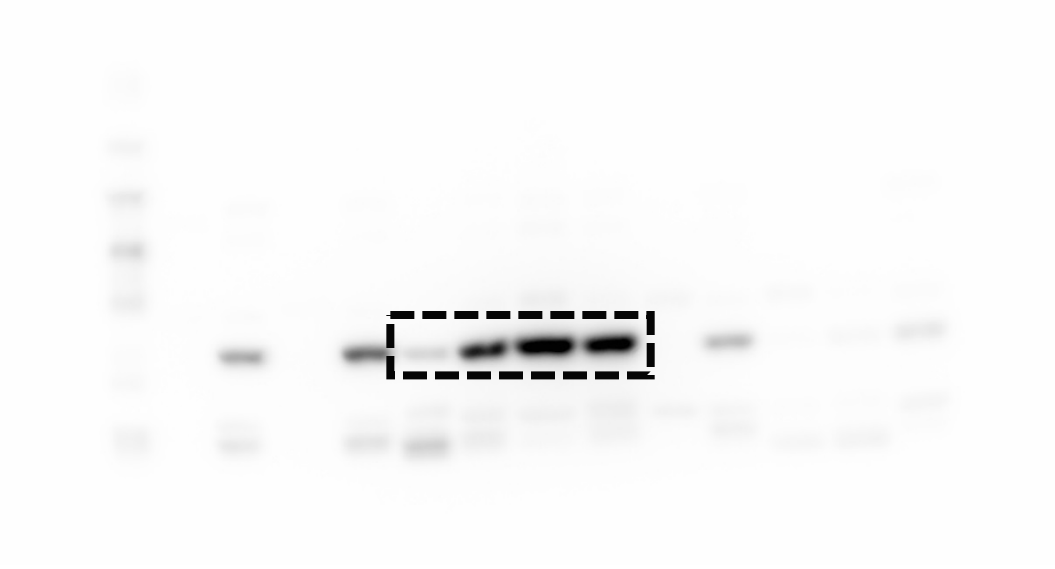

H3A

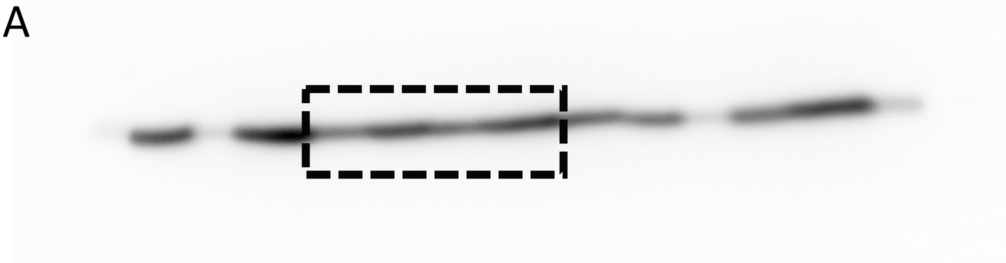

Supplemental Fig. 3

β-Actin

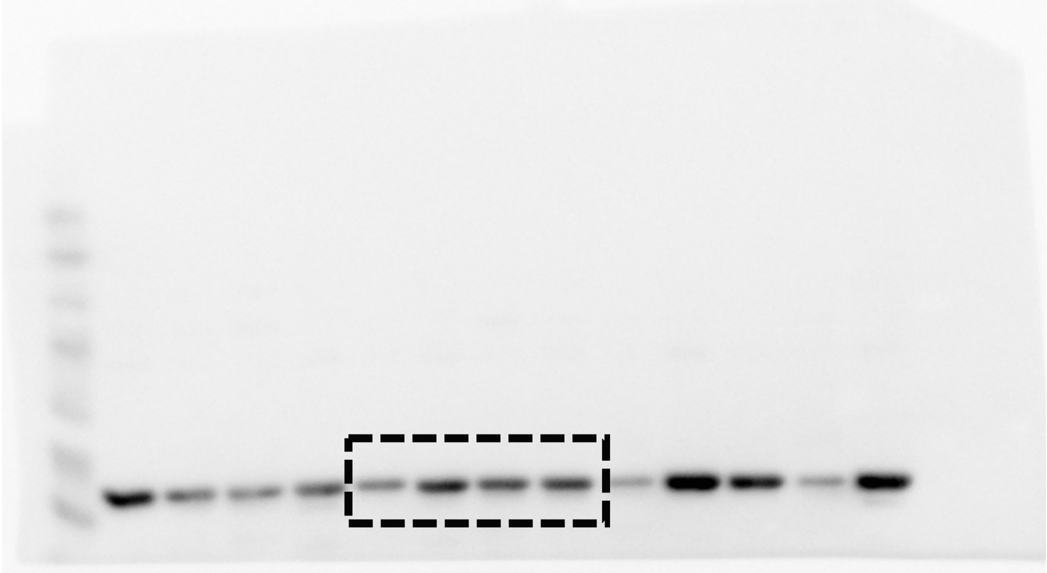

H3A

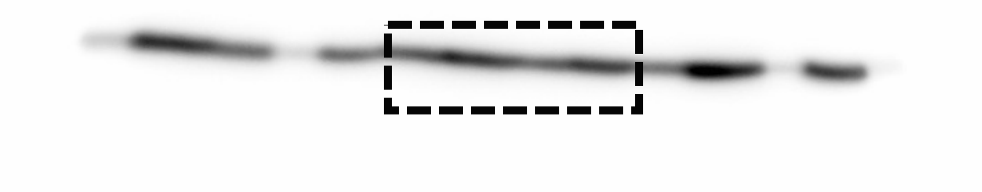

Supplemental Fig. 4

Myo-1a

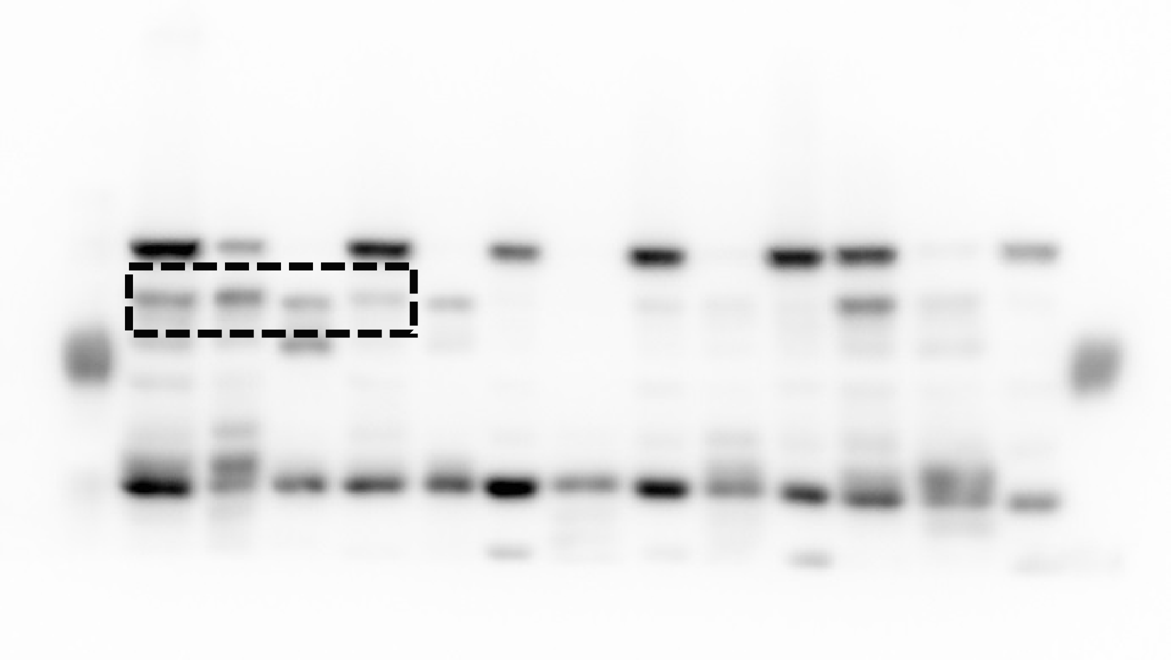

H3A

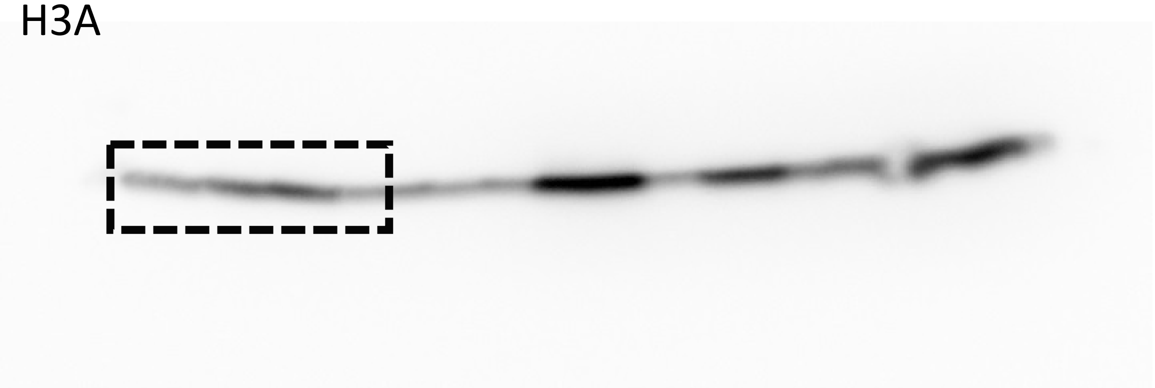

Supplemental Fig. 5

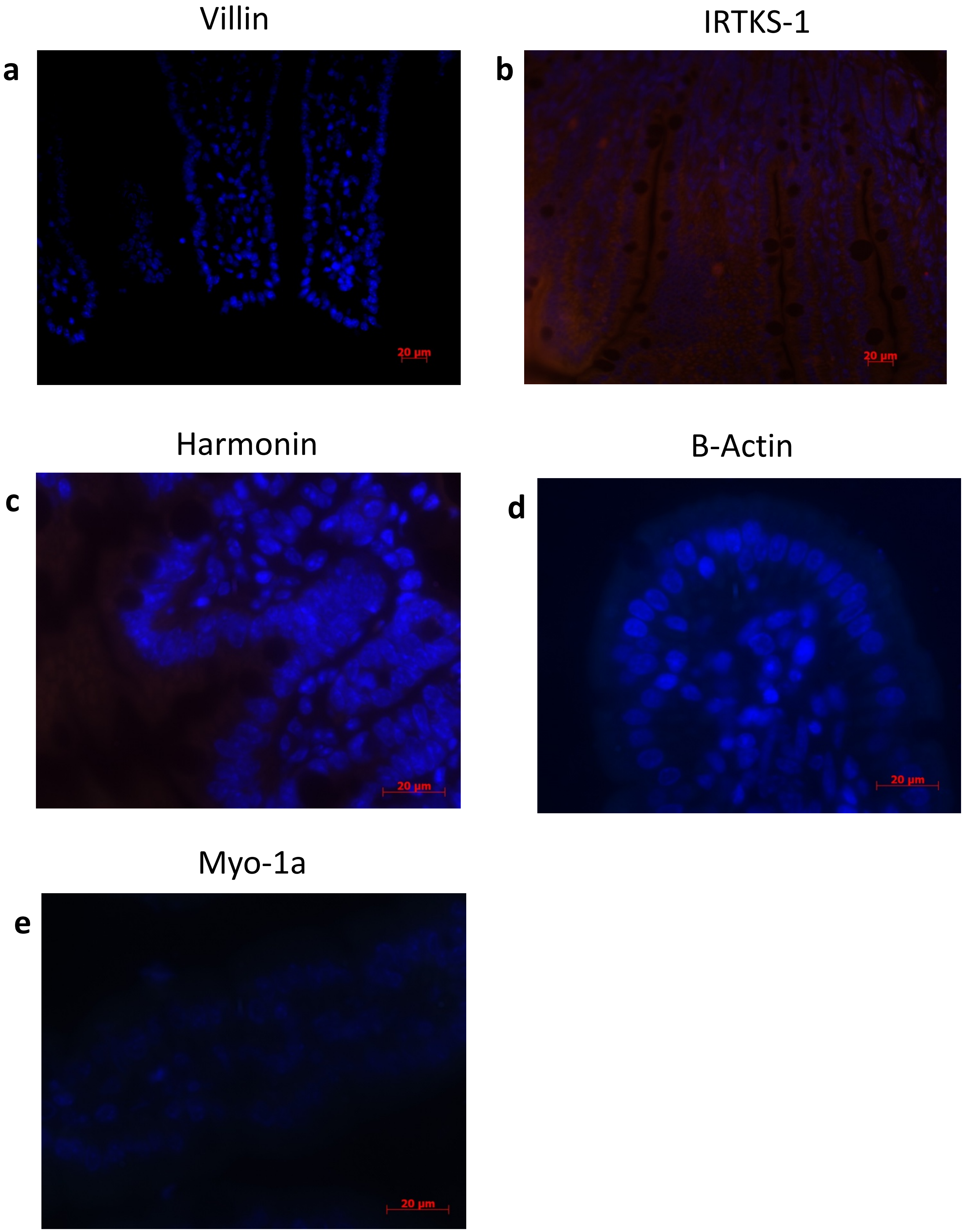

Supplemental Fig. 6
